# Red-brown and purpuric eroded papules and patches in the intertriginous regions

**DOI:** 10.1016/j.jdcr.2025.09.038

**Published:** 2025-10-11

**Authors:** Jeffery Hu, Stephanie Mengden-Koon, Kevin P. White, Angela J. Jiang

**Affiliations:** Department of Dermatology, Oregon Health and Sciences University, Portland, Oregon

**Keywords:** adult, intertriginous dermatoses, Langerhans cell histiocytosis

## Case

**Fig 1:**
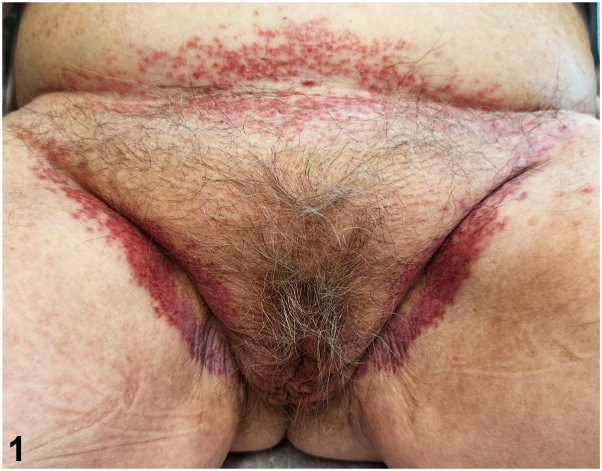

**Fig 2:**
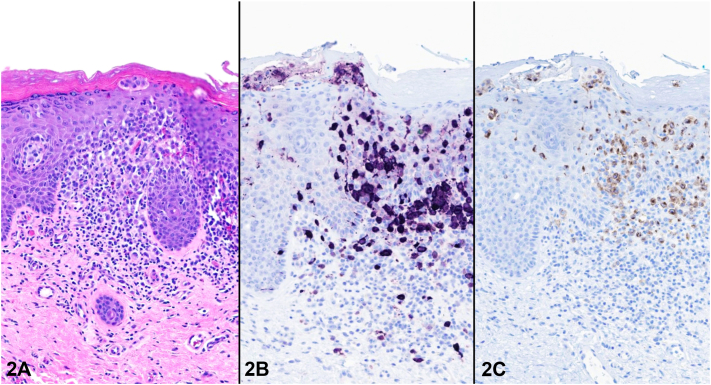

An 85-year-old female presented with a pruritic, intertriginous rash present for 5 years. She was initially treated with topical steroids and topical antifungals without improvement. She denied other symptoms. Physical examination revealed erythematous and purpuric confluent patches in the inner thighs, with some sparing of the inguinal folds (Fig 1). In the infrapannus, axillae, and inframammary regions, there were similar erythematous and purpuric eroded papules. A punch biopsy demonstrated an epidermotropic infiltrate of enlarged cells (Fig 2, *A*) that were positive for S100 and Langerin (Fig 2, *B* and *C*).

**Question 1: What is the diagnosis?**

A. Hailey-Hailey disease
B. Extramammary Paget disease
C. Langerhans cell histiocytosis (LCH)
D. Inverse psoriasis
E. Intertrigo

**Answers:**

A. Hailey-Hailey disease – Incorrect. Hailey-Hailey disease typically begins as flaccid vesicles and evolves into eroded plaques with linear fissures. Biopsies of Hailey-Hailey disease demonstrate suprabasilar acantholysis.
B. Extramammary Paget disease – Incorrect. Extramammary Paget disease classically presents as well-demarcated erythematous plaques with scattered erosions. The histopathology shows enlarged cells with pale cytoplasm that are highlighted by CK7, CAM5.2, and CEA, and are negative for S100.
C. LCH – Correct. In addition to the petechial and crusted appearance of the lesions, the histopathology demonstrating enlarged tumor cells with S100 and Langerin positivity and epidermotropism confirms the diagnosis.
D. Inverse psoriasis – Incorrect. Clinically, inverse psoriasis presents as well-demarcated patches with maceration and minimal scale. Biopsies demonstrate psoriasiform epidermal hyperplasia with hypogranulosis and thinning of the suprapapillary plates. Neutrophils are common within the stratum corneum and occasionally within the epidermis.
E. Intertrigo – Incorrect. Intertrigo typically presents as erythema of opposing skin surfaces with erosions or fissures. Biopsies of intertrigo are often non-specific but may show mild spongiosis.

**Question 2: What are the systemic considerations?**

A. Increase prevalence of metabolic syndrome
B. Unifocal or extensive systemic involvement with pulmonary, skeletal, and endocrine involvement being the most common
C. Concern for invasive adenocarcinoma
D. Psoriatic arthritis
E. No systemic concerns

**Answers:**

A. Increase prevalence of metabolic syndrome – Incorrect. Inverse psoriasis can be associated with an increased prevalence of metabolic syndrome.
B. Unifocal or extensive systemic involvement with pulmonary, skeletal, and endocrine involvement being the most common – Correct. LCH can have multisystem involvement.
C. Concern for invasive adenocarcinoma – Incorrect. Extramammary Paget disease would raise concern for an invasive adenocarcinoma.
D. Psoriatic arthritis – Incorrect. Inverse psoriasis may also raise consideration for psoriatic arthritis.
E. No systemic concerns – Incorrect. Hailey-Hailey does not have other systemic associations.

## Discussion

LCH is a rare neoplastic disorder that presents more often in children, and the pathogenesis of LCH is not yet understood.^1^ Adult-onset LCH is rare, with an estimated incidence of 1 to 2 per million adults.^1^ Pulmonary, bone, and endocrine involvement are the most common presentations in adulthood, but skin may be affected in 25% to 40% of cases.^2^ Isolated cutaneous involvement is rare and estimated to occur in 5% to 10% of cases.^2^ The clinical presentation can be variable, with the most common presentation being red-brown eroded and crusted papules on the scalp, trunk, skin folds, or groin.^2^ Other presentations may include a solitary tumor or ulcerating lesions in the skin folds.^3^ Histopathologic examination classically demonstrates a proliferation of intermediate-sized cells with reniform nuclei in the papillary dermis, which may be admixed with eosinophils.^2^ LCH cells demonstrate immunostaining positive for S100, CD1a, and Langerin (CD207).

For patients presenting to dermatology with cutaneous involvement of LCH, dermatologists should evaluate not only for systemic involvement of LCH, but also a potential secondary hematologic malignancy. There is an estimated incidence between 7% and 27% of adult patients presenting with cutaneous LCH developing a secondary hematologic malignancy.^3^^,^^4^ While there are no specific guidelines for staging and follow-up of adult-onset LCH, baseline imaging and close clinical and laboratory follow up is recommended. Some authors suggest positron emission tomography imaging, baseline laboratory monitoring (including complete blood count with differential, comprehensive metabolic panel, C-reactive protein), endocrine testing (including thyroid stimulating hormone, free T4, morning urine and serum osmolality, morning serum cortisol), and a bone marrow biopsy in patients presenting with cutaneous LCH.^2^^,^^3^

Localized treatment options include topical and intralesional steroids, topical calcineurin inhibitors, topical nitrogen mustard, imiquimod, phototherapy, radiation therapy, and surgical excision.^3^^,^^4^ Systemic treatments for skin-limited LCH may include systemic steroids, methotrexate, retinoids, thalidomide, vinblastine, interferon, and caldribine.^3^^,^^4^

## Conflicts of interest

None disclosed.
